# Henoch Schonlein Purpura as a Cause of Renal Failure in an Adult

**DOI:** 10.1155/2016/7890379

**Published:** 2016-09-08

**Authors:** Shweta Kukrety, Pradeepa Vimalachandran, Rajesh Kunadharaju, Vishisht Mehta, Agnes Colanta, Mahmoud Abu Hazeem

**Affiliations:** ^1^Department of Internal Medicine, Creighton University Medical Center, Omaha, NE, USA; ^2^Department of Family Medicine, Creighton University Medical Center, Omaha, NE, USA; ^3^Department of Pathology, Creighton University Medical Center, Omaha, NE, USA

## Abstract

Henoch Schonlein purpura (HSP) is an immune mediated disease associated Immunoglobulin A (IgA) deposition within the affected organs. While the disease is commonly seen in the pediatric age group, it is rarely seen in adults. We report the case of a 93-year-old Caucasian lady who presented with nonthrombocytopenic purpuric rash and acute kidney injury after an episode of bronchitis. Rapid and progressive deterioration of renal function prompted a kidney biopsy, which showed findings consistent with IgA nephropathy confirming the diagnosis of HSP. The patient was treated with high dose intravenous methylprednisolone followed by oral prednisone; however, her kidney disease progressed to end stage renal disease requiring hemodialysis. HSP is usually a self-limiting disease in children. However, adults are at an increased risk of severe renal involvement including end stage renal disease. Purpuric skin rash with renal involvement should raise suspicion for HSP. This is the oldest known patient with HSP.

## 1. Introduction

Henoch Schonlein purpura, also known as Immunoglobulin A vasculitis (IgAV) is a small vessel vasculitis associated with IgA deposition in the affected organs. HSP is primarily a disease of childhood with a peak incidence between 4 and 6 years of age [1]. In contrast, it is rarely seen in adults with an incidence of 0.1–1.2 per million in adults over 20 years of age [2]. The American College of Rheumatology has identified 4 criteria for the diagnosis of HSP: age less than or equal to 20 years at disease onset, palpable purpura, acute abdominal pain, and biopsy showing granulocytes in the walls of small arterioles or venules. The presence of any 2 or more of these criteria distinguishes HSP from other forms of vasculitis with a sensitivity of 87.1% and a specificity of 87.7% [3]. Management guidelines for HSP nephritis in an adult remain obscure and more research is needed in this area.

## 2. Case Presentation

Our patient was a 93-year-old Caucasian lady who presented with a three-day history of right upper quadrant abdominal pain, nausea, and vomiting. She had an episode of acute bronchitis two weeks before she presented to us, which was treated with Levofloxacin. A week after initiation of treatment for her acute bronchitis, the patient developed a purpuric rash involving both her lower extremities, which gradually progressed to involve her upper extremities as well. Upon further questioning, the patient revealed she had an episode of painless hematuria three days prior to the current presentation.

Her past medical history was significant for four-vessel coronary artery bypass grafting (CABG) in 1995, chronic atrial fibrillation on anticoagulation with warfarin, and hypertension and chronic kidney disease stage 3 with baseline creatinine in 1.3–1.5 range. Social history was unremarkable.

Physical exam revealed RUQ tenderness and a purpuric rash involving both upper and lower extremities.

Initial laboratory analysis showed leukocytosis with a white blood cell count of 21.2 k/mcl and creatinine of 3.39 mg/dL. Urinalysis showed hematuria and proteinuria with negative nitrites and leucocyte esterase. Urine eosinophils were negative. Liver function tests, lactic acid, procalcitonin, and cardiac enzymes were all within normal limits. An ultrasound of the abdomen showed findings suggestive of acute cholecystitis and no abnormalities in the renal anatomy. The general surgery team was consulted and the patient underwent a laparoscopic cholecystectomy.

**Figure 1:**
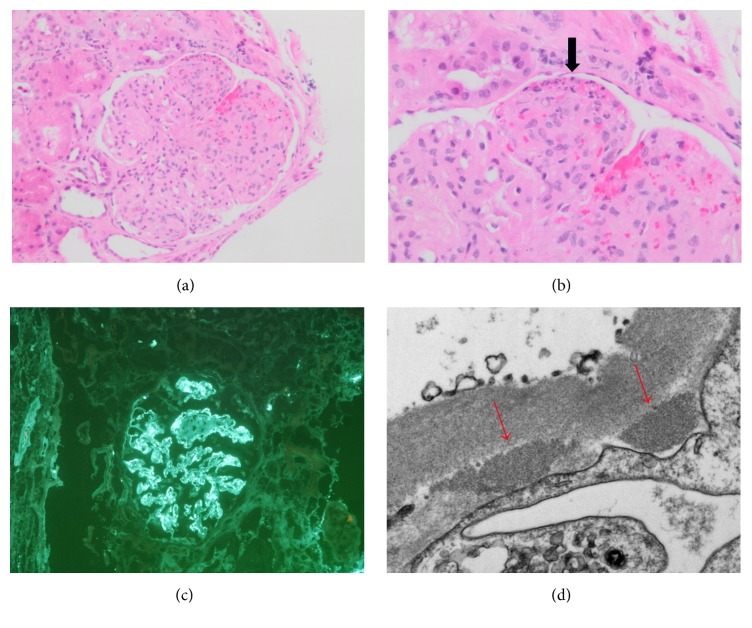
(a) Glomerulus with nodularity, mesangial hypercellularity, and increased mesangial matrix; no crescents were identified (H&E 200x). (b) A segment of the glomerulus showing fibrinoid necrosis and karyorrhexis (arrow) (H&E 400x). (c) Immunofluorescence microscopy showed strong granular IgA staining of mesangial regions and capillary walls (200x). (d) Electron microscopy showed subendothelial electron-dense deposits (arrows) (8000x).

The patient recovered well from her cholecystectomy and did not have any more episodes of abdominal pain; however, her kidney function continued to worsen progressively with creatinine peaking at 4.97 mg/dL. Her urine output became minimal and she began to show signs of volume overload on exam. An extensive work-up was done to evaluate the cause of her renal failure. Antinuclear antibodies (ANA), anti-neutrophil cytoplasmic antibodies (ANCA), anti-glomerular basement membrane antibody (anti-GBM Ab), serology for hepatitis B and C, and antistreptolysin O titres were all negative. Complement levels (C3 and C4) were low. IgA levels were normal. Rapid and progressive deterioration of the renal function prompted a renal biopsy, which showed an IgA nephropathy. A diagnosis of adult onset HSP was made based on the presence of nonthrombocytopenic purpura with renal biopsy showing IgA nephropathy (Figure 1).

The patient was initiated on high dose intravenous methylprednisolone for 3 days followed by oral prednisone, but her renal function continued to worsen eventually requiring hemodialysis. Patient also developed multilobar pneumonia with hypoxemic respiratory failure. After much discussion with the patient and family, the patient did not want further dialysis and was discharged home on hospice. She passed away within a few days.

## 3. Discussion

HSP is a small vessel IgA vasculitis seen predominantly in the pediatric age group. It is infrequently seen in adults with an incidence of 0.1–1.2 per million in adults over 20 years of age [2]. HSP shows a male predominance with a male to female ratio of 1.2–1.8 : 1 [1]. Its exact etiology is unknown, but a preceding infection may appear to play a role.

The classical tetrad of HSP includes palpable purpura without thrombocytopenia and coagulopathy, arthritis/arthralgia, abdominal pain, and renal involvement. Important differences in manifestation between adults and children are that adults are at an increased risk for significant renal involvement including end stage renal disease [4, 5].

Although HSP is an IgA mediated vasculitis, elevated IgA levels are seen in only 50–70% of the cases. Normal IgA levels are seen more commonly in adults [4]. Our patient demonstrated normal IgA levels. Serum complement levels are normal in most patients with HSP; however, hypocomplementemia is not unusual in HSP. It is generally a transient phenomenon and does not correlate with the severity of the disease [6]. Our patient also presented with low complement levels.

Diagnosis of HSP in the pediatric population is usually a clinical one; biopsy is usually reserved for unusual presentations. In the adult population, biopsy of the affected organ (skin or kidney) may be required for confirmation of the diagnosis due to much lower incidence in this population. HSP in children is usually a self-limiting disease and management is mainly supportive.

In adults, renal involvement tends to be more severe with progression to end stage renal disease. Kidney manifestations may be seen within a few days to one month after the onset of systemic symptoms.

Management of HSP nephritis remains controversial. Many different therapies have been reported to be beneficial in small case series in children; however, there is no evidence from controlled trials on any of these management strategies in adults. Niaudet and Habib recommended treatment with intravenous pulse methylprednisolone (3 days) followed by oral prednisone (3.5 months) [7]. Our patient was managed with intravenous pulse steroids followed by oral prednisone; however, her renal disease continued to progress and she had to be initiated on hemodialysis. A retrospective analysis by Park et al. in 29 children showed that cyclosporine may be beneficial in patients with nephrotic range proteinuria [8]. Other small uncontrolled studies have shown benefit with glucocorticoids with azathioprine [9], high dose IV immunoglobulin therapy [10], and plasmapheresis [11]. However, more data is required from controlled trials to confirm benefit. Renal transplant may be an option in patients who progress to end stage renal disease, although HSP nephritis frequently recurs after renal transplantations [12].

## 4. Conclusion

Diagnosis of HSP in the adult patient can be challenging and needs to be differentiated from other causes of vasculitis. Palpable purpura in the absence of thrombocytopenia or coagulopathy with renal involvement should raise the suspicion of HSP. Adults frequently present with renal manifestations, which tend to be severe. There are no clear guidelines on management of HSP nephritis, although many different therapies have been reported to be beneficial. Further evidence is required from randomised control trials to prove benefit.
